# Supplementary material for: Ferroptosis Inducers Kill Mesenchymal Stem Cells Affected by Neuroblastoma
Source: Cancers (Basel). 2023 Feb 18;15(4):1301. doi: 10.3390/cancers15041301 (PMC9954189; doi:10.3390/cancers15041301)
Supplement: Supplementary file 1 [file cancers-15-01301-s001.zip › cancers-2182820-supplementary.pdf]

Supplementary data

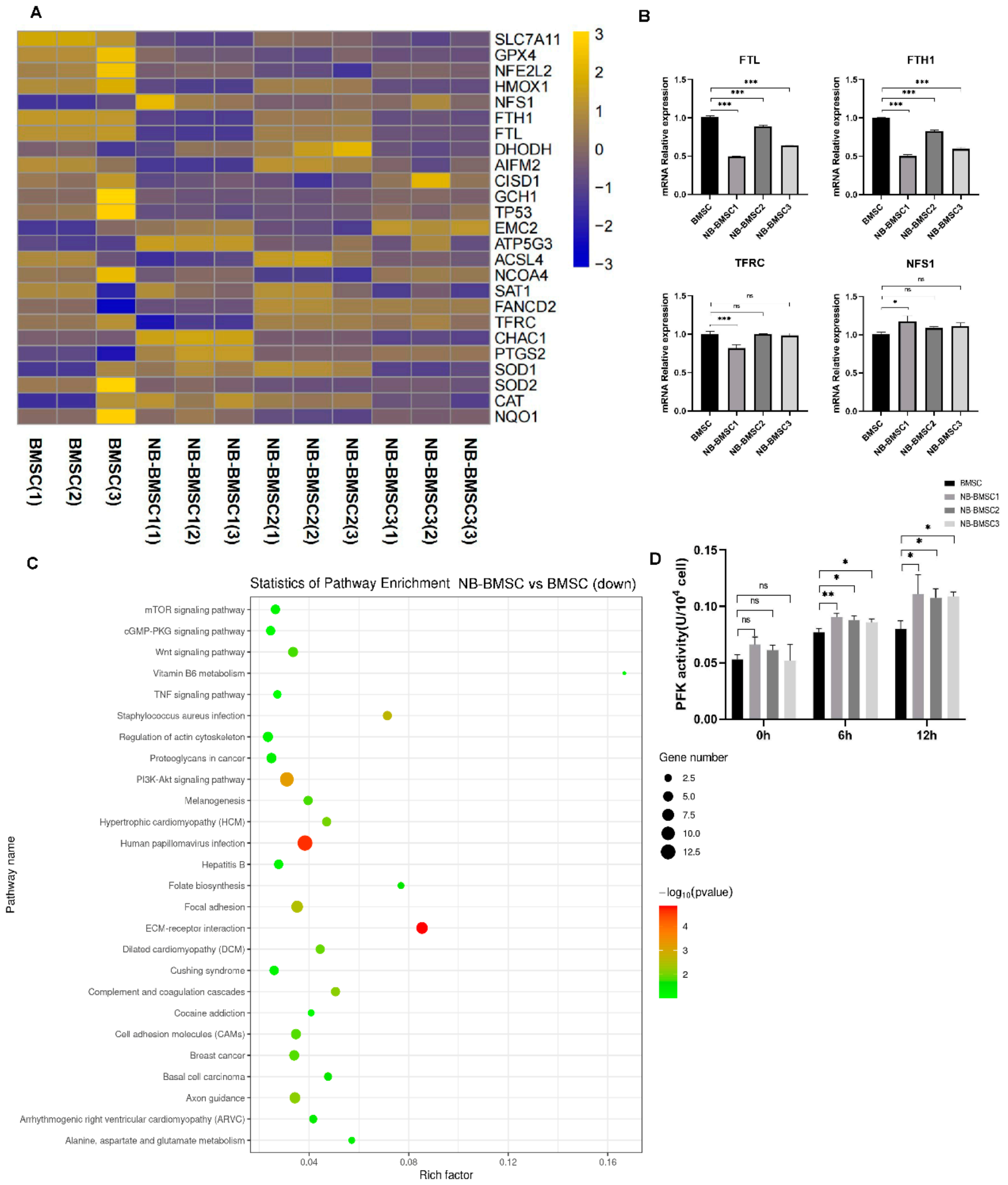

Figure S1: Details of RNA sequencing were shown. (**A, B**) The single result of RNA sequencing and the RNA expression related to this article. (**C**) The pathways downregulated in NB-BMSCs were analysed. (**D**) PFK activity of BMSC and NB-BMSC was detected after H2O2 treatment. A one-way analysis of variance was used. ^{ns} not significant; * $p < 0.05$; ** $p < 0.01$; *** $p < 0.001$.

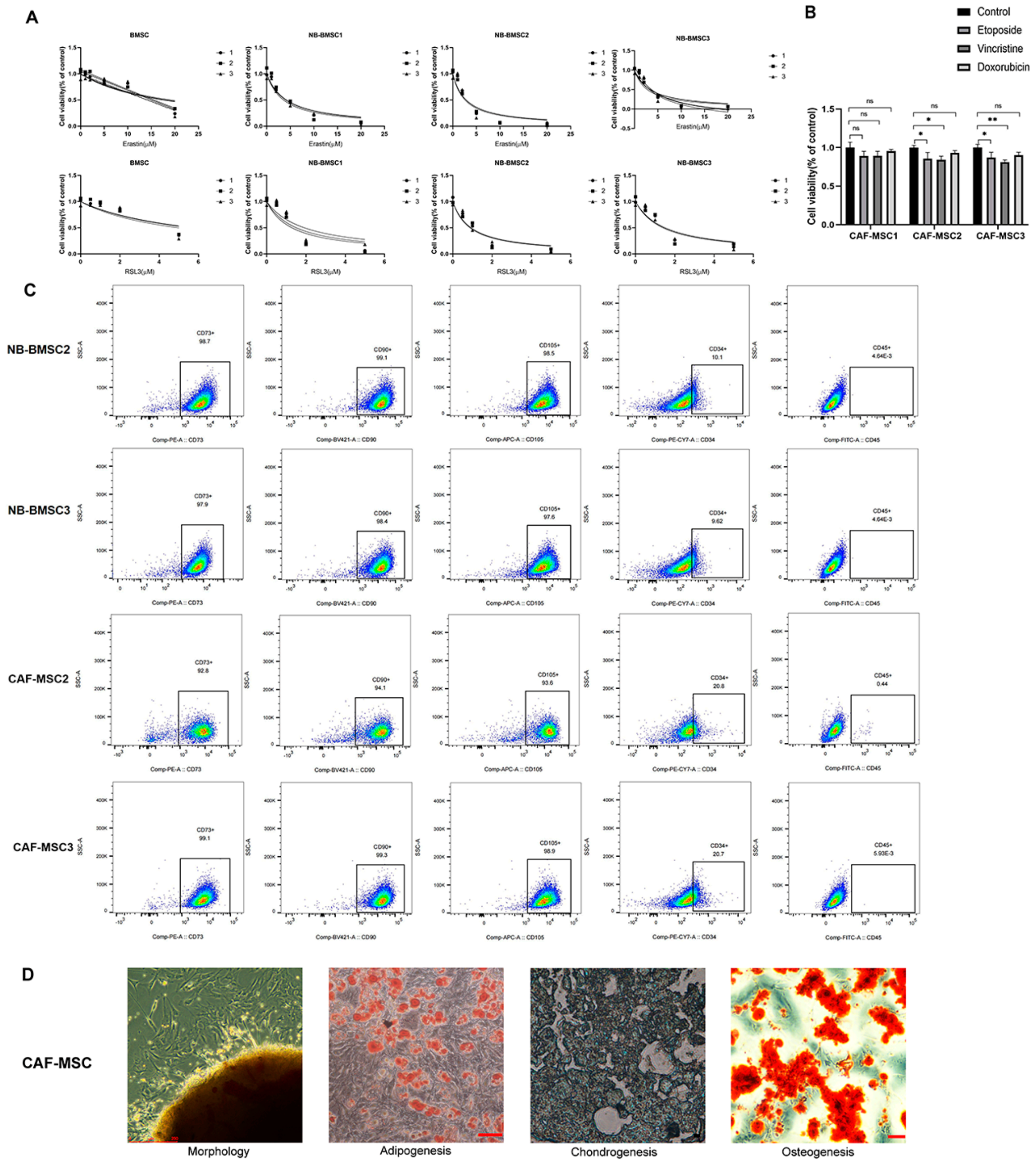

Figure S2: Etoposide, vincristine or doxorubicin can't kill CAF-MSCs from patients effectively. (**A**) The experiments on the basis of which the IC50 values for erastin and RSL3 were calculated. (**B**) Cell viability of CAF-MSCs was detected under etoposide (10μM), vincristine (10μM) or doxorubicin (20 μM). (**C**) FACS analysis for detecting surface markers of all of NB-BMSCs and CAF-MSCs from NB patients. (**D**) Morphology, adipogenic, chondrogenic, and osteogenic differentiation potentials of CAF-MSCs from NB patients. Scale bar: 200 μm. A one-way analysis of variance was used. ^{ns} not significant; * $p < 0.05$; ** $p < 0.01$.

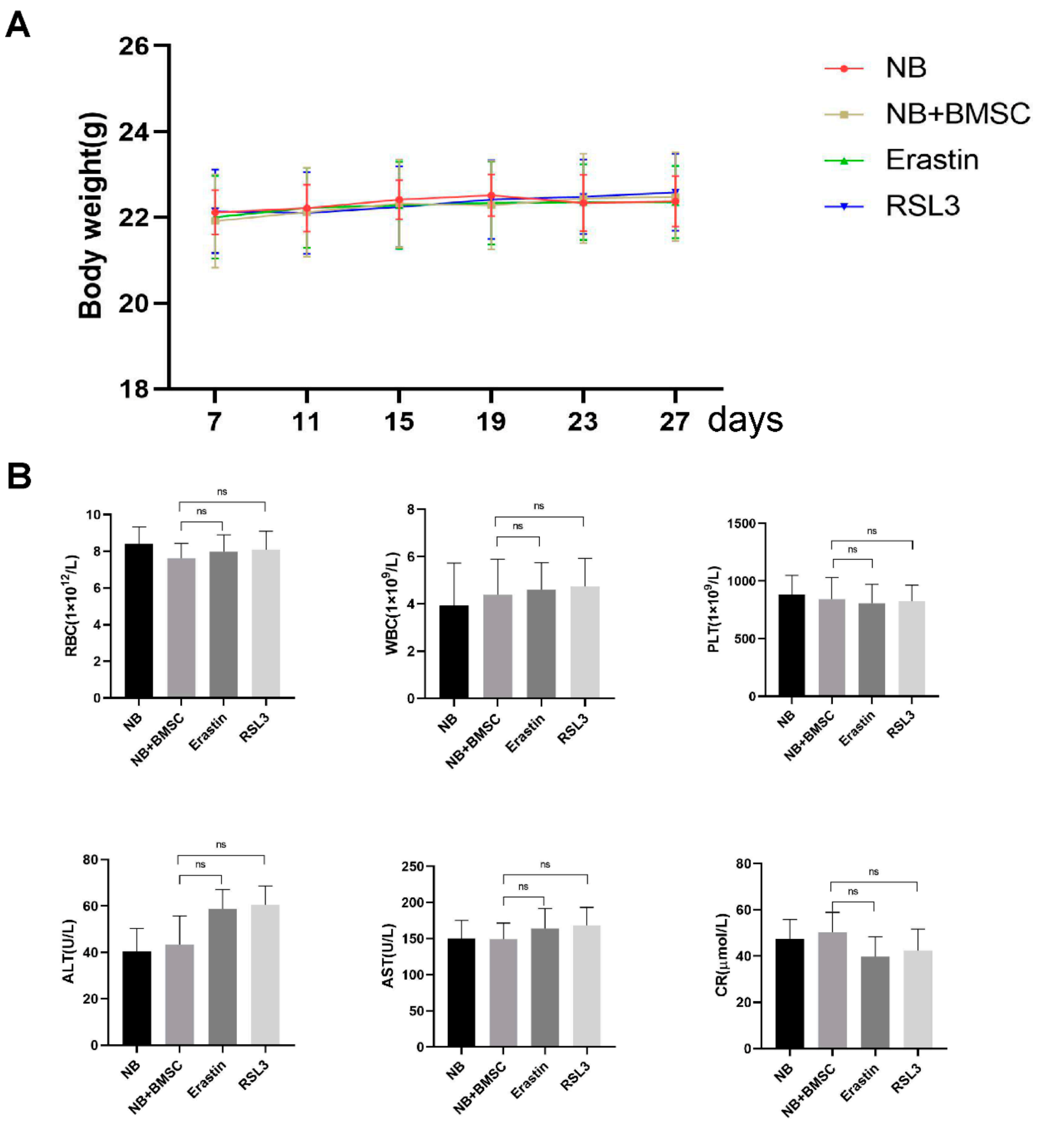

Figure S3: Details of body weight, blood routine and blood biochemistry were shown. A one-way analysis of variance was used. [ns] not significant.

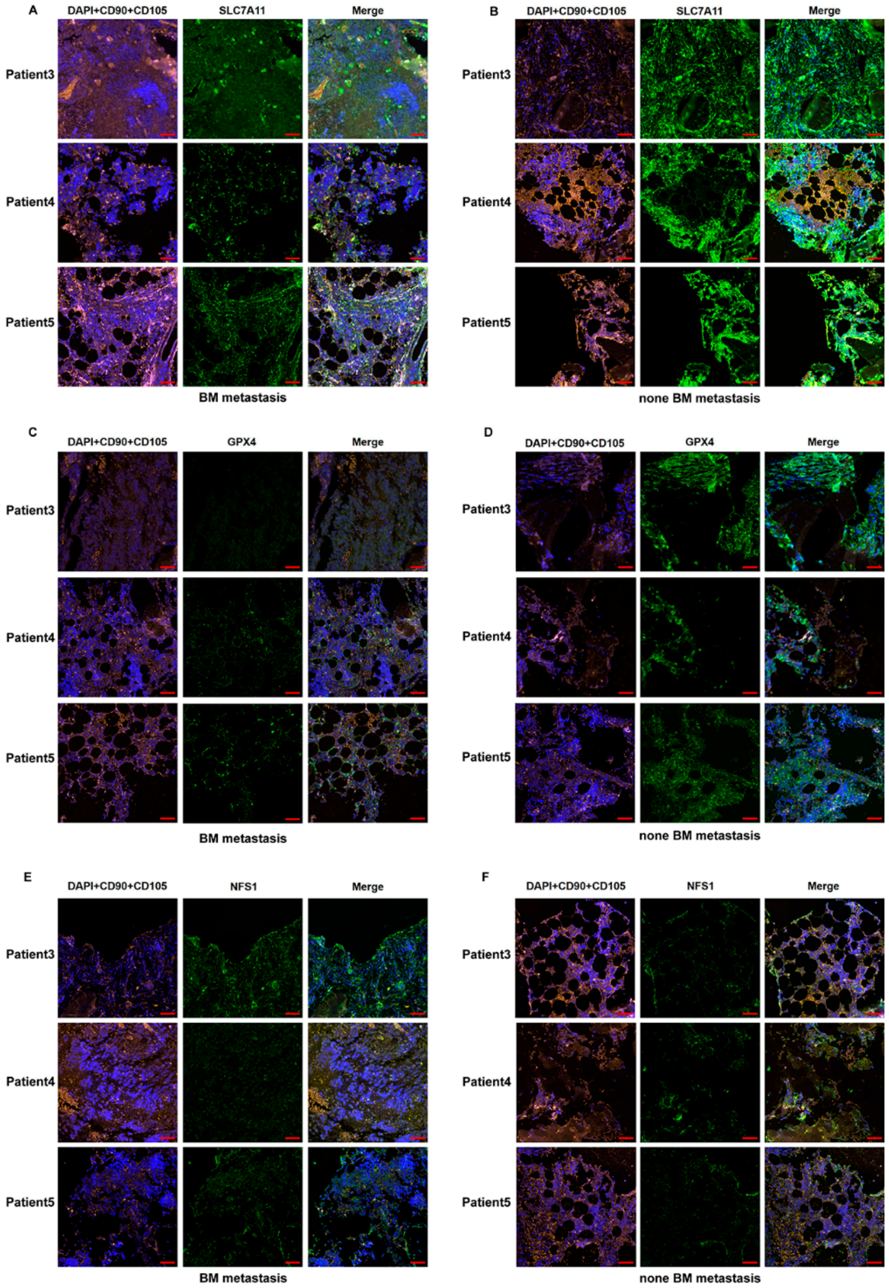

Figure S4: Immunofluorescence of BMSCs in patients with or without BM metastasis. Scale bar: 50 μm.
